# Histone deacetylase turnover and recovery in sulforaphane-treated colon cancer cells: competing actions of 14-3-3 and Pin1 in HDAC3/SMRT corepressor complex dissociation/reassembly

**DOI:** 10.1186/1476-4598-10-68

**Published:** 2011-05-30

**Authors:** Praveen Rajendran, Barbara Delage, W Mohaiza Dashwood, Tian-Wei Yu, Bradyn Wuth, David E Williams, Emily Ho, Roderick H Dashwood

**Affiliations:** 1 Linus Pauling Institute, Oregon State University, Weniger 503, Corvallis, OR 97331-6512, USA; 2 Dept. Nutrition and Exercise Sciences, Oregon State University, Milam 117, Corvallis, OR 97331-5130, USA; 3 Dept. Environmental and Molecular Toxicology, ALS 1007, Oregon State University, Corvallis, OR 97331, USA

## Abstract

**Background:**

Histone deacetylase (HDAC) inhibitors are currently undergoing clinical evaluation as anti-cancer agents. Dietary constituents share certain properties of HDAC inhibitor drugs, including the ability to induce global histone acetylation, turn-on epigenetically-silenced genes, and trigger cell cycle arrest, apoptosis, or differentiation in cancer cells. One such example is sulforaphane (SFN), an isothiocyanate derived from the glucosinolate precursor glucoraphanin, which is abundant in broccoli. Here, we examined the time-course and reversibility of SFN-induced HDAC changes in human colon cancer cells.

**Results:**

Cells underwent progressive G_2_/M arrest over the period 6-72 h after SFN treatment, during which time HDAC activity increased in the vehicle-treated controls but not in SFN-treated cells. There was a time-dependent loss of class I and selected class II HDAC proteins, with HDAC3 depletion detected ahead of other HDACs. Mechanism studies revealed no apparent effect of calpain, proteasome, protease or caspase inhibitors, but HDAC3 was rescued by cycloheximide or actinomycin D treatment. Among the protein partners implicated in the HDAC3 turnover mechanism, silencing mediator for retinoid and thyroid hormone receptors (SMRT) was phosphorylated in the nucleus within 6 h of SFN treatment, as was HDAC3 itself. Co-immunoprecipitation assays revealed SFN-induced dissociation of HDAC3/SMRT complexes coinciding with increased binding of HDAC3 to 14-3-3 and peptidyl-prolyl cis/trans isomerase 1 (Pin1). Pin1 knockdown blocked the SFN-induced loss of HDAC3. Finally, SFN treatment for 6 or 24 h followed by SFN removal from the culture media led to complete recovery of HDAC activity and HDAC protein expression, during which time cells were released from G_2_/M arrest.

**Conclusion:**

The current investigation supports a model in which protein kinase CK2 phosphorylates SMRT and HDAC3 in the nucleus, resulting in dissociation of the corepressor complex and enhanced binding of HDAC3 to 14-3-3 or Pin1. In the cytoplasm, release of HDAC3 from 14-3-3 followed by nuclear import is postulated to compete with a Pin1 pathway that directs HDAC3 for degradation. The latter pathway predominates in colon cancer cells exposed continuously to SFN, whereas the former pathway is likely to be favored when SFN has been removed within 24 h, allowing recovery from cell cycle arrest.

## Background

Epigenetic changes play a critical role in cancer development [1-5]. These changes include the dysregulation of histone deacetylases (HDACs) and the altered acetylation status of histone and non-histone proteins [6-8]. Efforts have been directed at reversing aberrant acetylation patterns in cancers through the use of HDAC inhibitors. HDAC inhibitors induce cell cycle arrest, differentiation, and apoptosis in cancer cells, some have anti-inflammatory activities, and a number have progressed to clinical trials [8-12].

HDACs can be overexpressed in colorectal cancers and in several other cancer types [13-18]. Silencing of HDACs, individually or in combination, has provided insights into the associated molecular pathways that regulate cell cycle transition, proliferation, and apoptosis [14,18-20]. In human colon cancer cells, silencing of HDAC3 resulted in growth inhibition, decreased cell survival, and increased apoptosis [14]. Similar effects were noted for HDAC2 and, to a lesser extent, for HDAC1. Subsequent work [18] identified a role for HDAC4 in regulating p21^WAF1 ^expression, via a corepressor complex involving HDAC4, HDAC3, and SMRT/N-CoR (silencing mediator for retinoid and thyroid hormone receptors/nuclear receptor co-repressor). Spurling *et al. *[16] reported that knockdown of HDAC3 increased constitutive, trichostatin A (TSA)-, and tumor necrosis factor (TNF)-α-induced expression of p21^WAF1^, although HDAC3 silencing alone did not account for all the gene expression changes observed upon general HDAC inhibition. Cells with lowered HDAC3 expression had increased histone H4-K12 acetylation (H4K12ac) and were poised for gene expression changes [16]. Ma *et al. *[20] observed that recruitment of p300 to the *survivin *promoter led to the concomitant recruitment of other protein partners, including HDAC6, resulting in transcriptional repression. Thus, there is accumulating evidence for the involvement of multiple HDACs in colon cancer development.

HDAC activity and histone acetylation status can be influenced by dietary factors and their metabolites [21-23]. For example, broccoli and broccoli sprouts are a rich source of glucoraphanin, the glucosinolate precursor of the cancer chemoprotective agent sulforaphane (SFN) [24-28]. SFN has been reported to inhibit HDAC activity in human colon cancer cells [29], and this was confirmed in prostate and breast cancer cells [30,31]. A structurally-related isothiocyanate also inhibited HDAC activity in human leukemia cells, resulting in chromatin remodeling and growth arrest [32]. Combining these findings with the changes induced by SFN in NF-E2-related factor 2 (Nrf2) signaling [24-28,33], a "one-two" chemoprotective model can be proposed. In the first stage, SFN parent compound induces phase 2 detoxification pathways, and in the second stage SFN metabolites alter HDAC activity and histone status, leading to the unsilencing of tumor suppressors such as p21^WAF1 ^[34-36]. An unresolved question from our earlier studies [29] was the fate of individual HDACs in SFN-treated colon cancer cells. If, indeed, SFN metabolites act as weak ligands for HDACs [37], does this result in de-recruitment and/or turnover of specific HDAC proteins, and is this reversible? These questions were examined in the present investigation, along with the molecular mechanisms involved.

## Results

### SFN-induced changes in HDAC activity and protein expression

**Figure 1:**
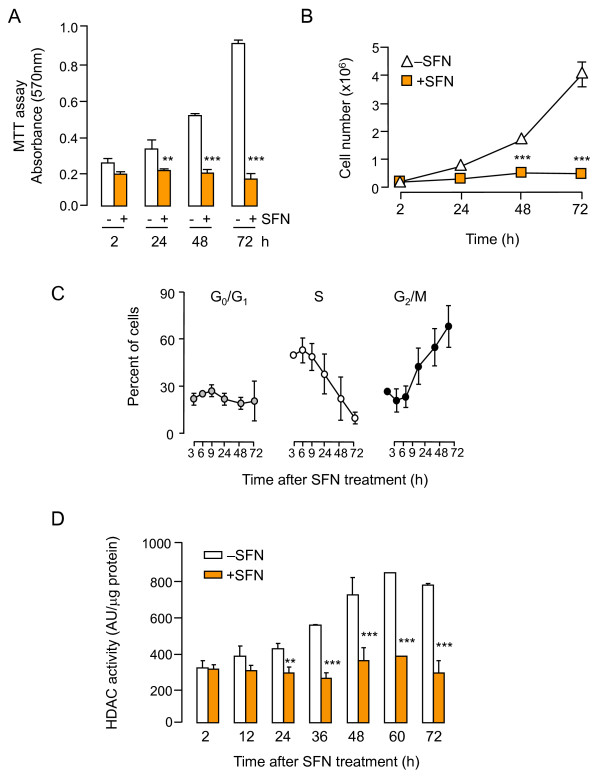
**Time-course studies of sulforaphane (SFN)-induced changes in cell cycle progression and histone deacetylase (HDAC) activity**. Human HCT116 colon cancer cells were plated at 0.1 × 10^6 ^cells/dish and 24 h later they were treated with SFN (15 μM), or with DMSO as vehicle control (-SFN). At selected times thereafter whole cell lysates were evaluated in the (A) MTT assay, (B) ViaCount assay, (C) Guava Cell Cycle Assay, and (D) HDAC activity assay (BioMol kit), as described in Methods. Data (mean ± SE, n = 3) were from a single experiment in each case, and are representative of the findings from three separate experiments. **P < 0.01; ***P < 0.001, compared with the corresponding vehicle control.

In our earlier studies in human colon cancer cells [29], the maximum concentration of SFN was 15 μM. Higher concentrations of SFN trigger extensive caspase-mediated apoptosis [38], and activated caspases can cleave HDACs [39,40]. Thus, unless stated otherwise, the nominal concentration of SFN used here was 15 μM. Under these conditions, vehicle-treated HCT116 human colon cancer cells exhibited a 4-fold increase in cell viability, whereas SFN-treated cells exhibited no changes for up to 72 h (Figure 1A). Over the same time-course, the cell number increased markedly for the vehicle controls, but remained constant for SFN-treated cells (Figure 1B). For the period 6-72 h post-SFN treatment, there was a dramatic increase in the proportion of cells occupying G_2_/M of the cell cycle, with a loss of cells in S phase (Figure 1C). Vehicle-treated cells grew rapidly and then arrested in G_0_/G_1_, 48-72 h post-treatment (data not shown). HDAC activity in whole cell lysates from vehicle-treated cells increased steadily and reached a plateau between 48-72 h (Figure 1D, open bars), whereas HDAC activity remained essentially unchanged in the SFN-treated cells. The difference in HDAC activity between vehicle- and SFN-treated cells was statistically significant at 24 h and time-points thereafter (Figure 1D). Similar time-course changes also were observed in HT29 colon cancer cells (data not presented).

**Figure 2:**
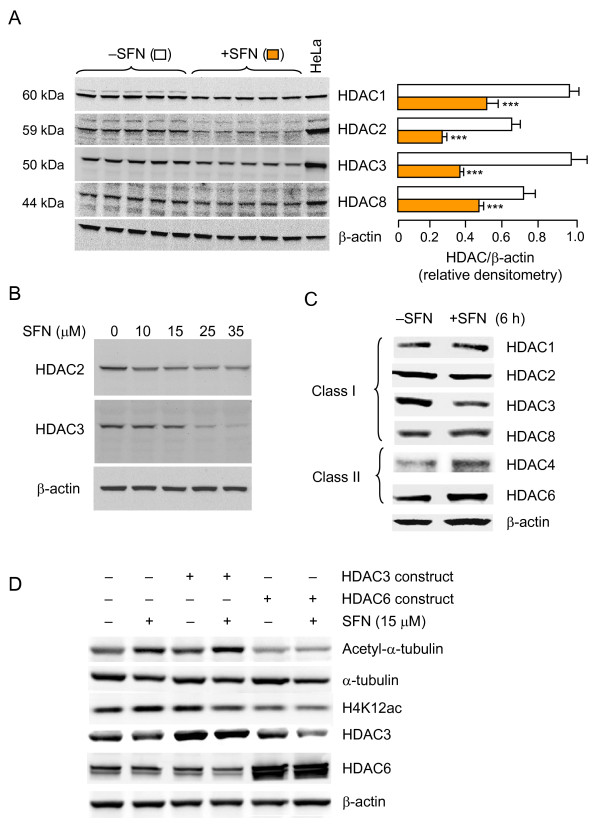
**Loss of HDAC protein expression in SFN-treated cells**. (A) HCT116 cells were treated as described in Figure 1 legend, except that five replicate plates were used for SFN and vehicle, respectively, and 36 h later class I HDACs were immunoblotted in whole cell lysates. Loading control, β-actin. HeLa nuclear extract was included as a reference. Right panel: HDAC expression normalized to β-actin (mean ± SE, n = 5), ***P < 0.001 for SFN *versus *the corresponding vehicle control. (B) Concentration-dependent loss of HDAC2 and HDAC3, 24 h post-SFN treatment. (C) Expression of class I and selected class II HDACs at 6-h post-SFN exposure. (D) Transient overexpression of HDAC6 and HDAC3 in HCT116 cells blocks tubulin hyperacetylation and/or histone H4K12 acetylation (H4K12ac) induced by SFN. Results are representative of the findings from two or more experiments.

The mid-point at 36 h was selected for immunoblotting studies of all four class I HDACs. Compared with the vehicle controls, there was a significant reduction in HDAC1, HDAC2, HDAC3 and HDAC8 protein expression in the SFN-treated cells (Figure 2A). Among the class I HDACs, HDAC3 was the most susceptible to SFN-induced loss of protein expression. For example, when cells were treated with 35 μM SFN and the whole cell lysates were immunoblotted at 48 h, HDAC2 was diminished by ~50% whereas HDAC3 was reduced by >95% (Figure 2B). HDAC3 also responded earliest to SFN treatment, the loss of protein expression being detected within 6 h, before the loss of other HDACs (Figure 2C). Among the class II HDACs, HDAC5, HDAC7, HDAC9 and HDAC10 were unchanged at all time-points up to 72 h (data not shown), whereas HDAC6 and HDAC4 proteins were reduced after 24 h (see below). Interestingly, transient overexpression of HDAC6, a tubulin-deacetylase [41,42], blocked not only the SFN-induced acetylation of tubulin, but also the SFN-mediated increase in H4K12ac (Figure 2D). Under the same experimental conditions, HDAC3 overexpression blocked the SFN-induced increase in H4K12ac without affecting tubulin acetylation status.

### Changes in HDAC protein expression are reversed upon SFN removal

**Figure 3:**
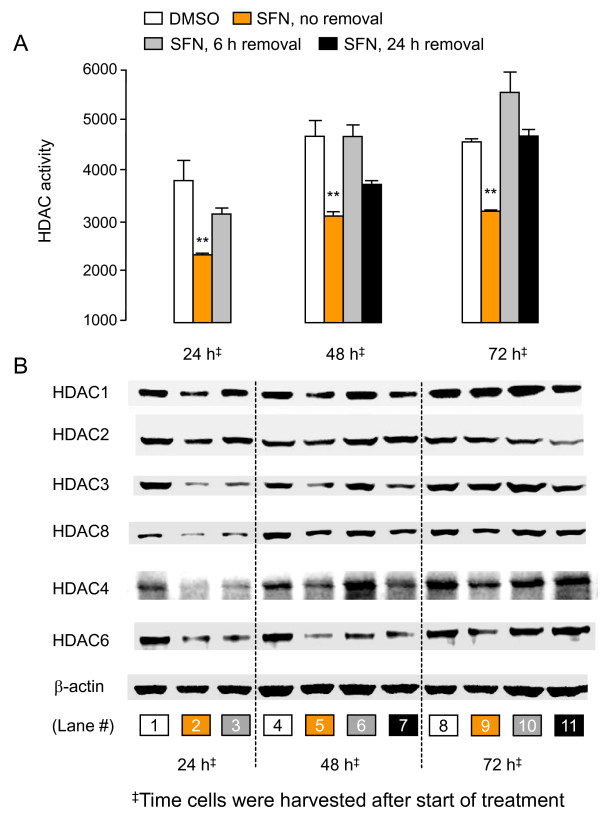
**Reversal of HDAC protein loss upon SFN removal**. (A) HCT116 cells were treated as described in Figure 1 legend, except that in some cases the SFN was removed after 6 or 24 h and replaced with fresh media containing no SFN. HDAC activity was determined for whole cell lysates obtained 24, 48 or 72 h after SFN was first added to the cells. Data (mean ± SE, n = 3) are from a single experiment, and are representative of the findings from three separate experiments. **P < 0.01 *versus *the corresponding DMSO control. (B) Whole cell lysates corresponding to the HDAC assay in (A) were immunoblotted for selected HDACs.

HCT116 cells were treated with 15 μM SFN and then SFN was removed 6 h or 24 h later and replaced with fresh media containing no SFN. Alternatively, SFN was added to the cells and left in the assay until harvest at 24, 48, or 72 h. When SFN was not removed and the cells were harvested at 24 h, as before, HDAC activity was significantly lower than in the vehicle controls (Figure 3A, top left, compare orange bar *versus *white bar, P < 0.01). However, in cells exposed to SFN for 6 h followed by SFN removal and addition of fresh media containing no SFN, HDAC activity at 24 h was no longer attenuated significantly (Figure 3A, top left, gray bar *versus *white bar).

The corresponding whole cell lysates were subjected to immunoblotting (Figure 3B). Expression levels of HDAC1, HDAC2, HDAC3, HDAC4, HDAC6, and HDAC8 were reduced when SFN was added to the assay and not removed, compared with the corresponding vehicle controls at 24 h (lane 2 *versus *lane 1, Figure 3B). When SFN was removed after 6 h and replaced with fresh media containing no SFN, there was complete recovery of HDAC1 and HDAC2 by 24 h, but no recovery of the other HDACs at this time-point (lane 3, Figure. 3B).

After a further 24 h, the HDAC activity had fully recovered in cells treated with SFN for 6 h (Figure 3A, 48 h, gray bar *versus *white bar), and there was complete recovery of all HDAC proteins, except HDAC6 (Figure 3B, compare lane 6 *versus *lane 4). Notably, even in cells exposed to SFN for 24 h followed by SFN removal, partial recovery of HDAC activity was detected by 48 h (Figure 3A, solid black bar). By 72 h, HDAC activity and protein expression had more-or-less fully recovered, except in cells treated continuously with SFN.

### Histone acetylation, cell cycle, and apoptosis changes upon SFN removal

**Figure 4:**
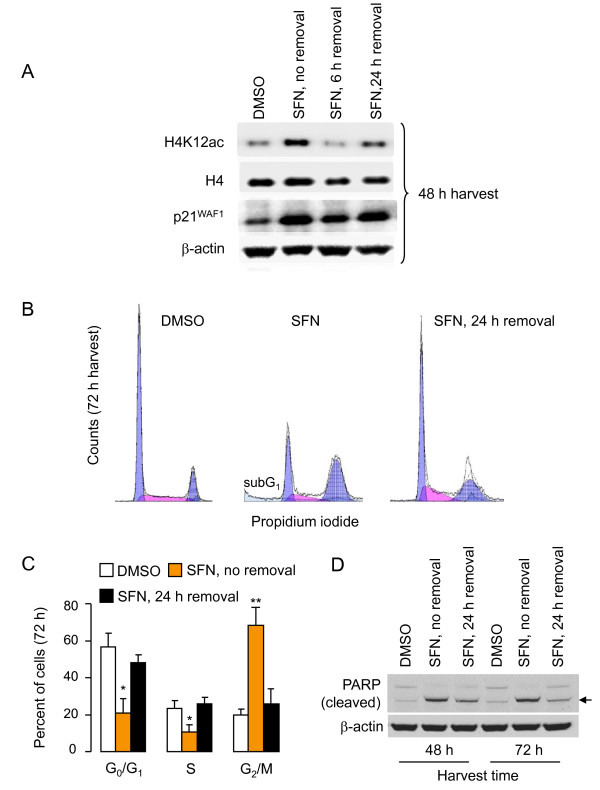
**Normalization of histone acetylation status and cell cycle progression upon SFN removal**. (A) HCT116 cells were treated with 15 μM SFN as described in Figure 3 legend, using 6-h, 24-h, and continuous exposure protocols. At 48 h after SFN was first added to the cells, whole cell lysates were prepared and subjected to immunoblotting for total histone H4 (H4), H4K12ac, p21^WAF1^, and β-actin. (B) The cell cycle distribution was determined after 72 h using flow cytometry (see Methods), for HCT116 cells treated with 15 μM SFN continually, or for 24 h and replaced with fresh media containing no SFN. (C) The experiment in (B) was repeated three times and the percent of cells in G_0_/G_1_, S, and G_2_/M was quantified. Data (mean ± SE, n = 3); *P < 0.05, **P < 0.01 *versus *the corresponding DMSO control. (D) HCT116 cells were treated with 15 μM SFN continually or for 24 h and replaced with fresh media (no SFN), and the corresponding whole cell lysates were immunoblotted at 48 or 72 h for full-length poly(ADP-ribose)polymerase (PARP), or its cleavage product (arrow). Results are representative of the findings from two or more separate experiments.

Subsequent experiments showed that histone hyperacetylation, p21^WAF1 ^induction, G_2_/M cell cycle arrest, and apoptosis induction were reversible upon SFN removal. Thus, HCT116 cells treated with SFN and harvested at 48 h, with no SFN removal, had increased H4K12ac and p21^WAF1 ^expression (Figure 4A). Upon removal of SFN at 6 h or 24 h and addition of fresh media containing no SFN, H4K12ac levels were completely or partially reversed. Normalizing to total histone H4 and β-actin, respectively, the relative order of H4K12 acetylation and p21^WAF1 ^induction was as follows: DMSO < SFN (6 h removal) < SFN (24 h removal) < SFN (no removal). As before (Figure 1C), with no SFN removal HCT116 cells arrested in G_2_/M, and eventually this was associated with the appearance of a subG_1 _population indicative of apoptosis (Figure 4B, middle panel). With SFN treatment for 24 h followed by removal and harvest at 72 h, few if any cells were detected in subG_1_, and most of the cells had escaped from G_2_/M arrest (Figure 4B, right panel). Quantification of three independent experiments confirmed that the cell cycle distribution was essentially no different between the vehicle controls and cells in which SFN had been removed after 24 h (Figure 4C, open *versus *solid black bars). Poly (ADP-ribose) polymerase (PARP) cleavage was evident at 48 h and 72 h in cells for which SFN had been added and not removed, but this was partially reversed when SFN was removed at 24 h and replaced with fresh media containing no SFN (Figure 4D).

### SFN-induced loss of HDAC3 is independent of caspase activity

**Figure 5:**
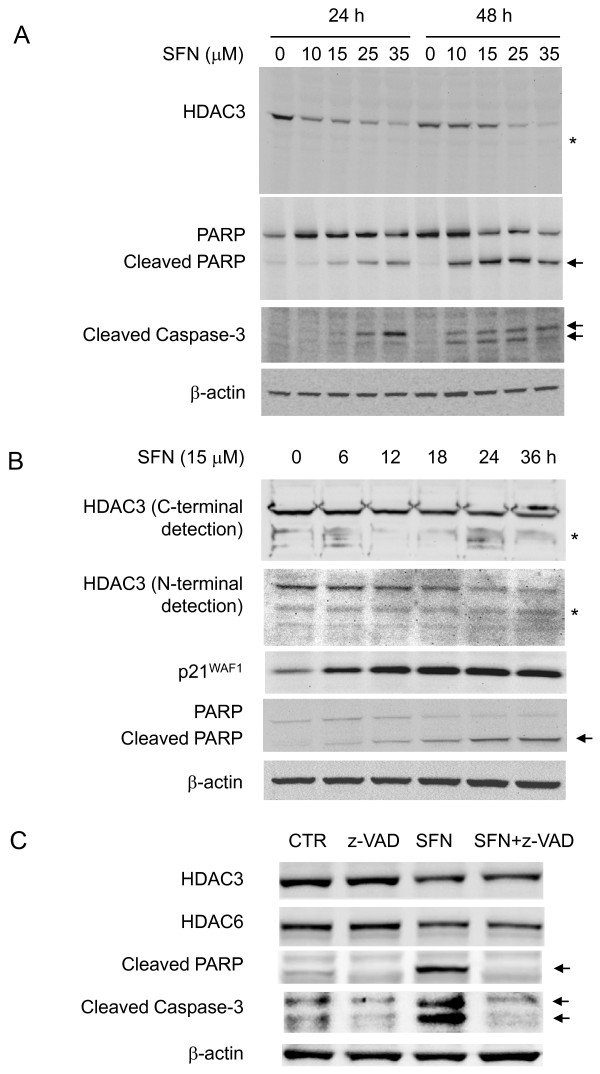
**SFN-induced HDAC3 loss is independent of caspase-3 activity**. (A) HCT116 cells were treated with various concentrations of SFN and the whole cell lysates were immunoblotted at 24 and 48 h for HDAC3, PARP/cleaved PARP, and cleaved (active) caspase-3. Asterisk, position of HDAC3 cleavage product reported by Escaffit *et al. *[39]; arrows, position(s) of the cleavage product(s) of PARP and caspase-3. (B) Loss of full-length HDAC3 detected with antibodies specific to the C- and N-terminal portions of the HDAC3 protein; no corresponding increase was detected for the HDAC3 cleavage product (asterisk). Whole cell lysates also were immunoblotted for p21^WAF1^and PARP. (C) HCT116 cells were treated with a cell-permeable pan caspase inhibitor (z-VAD(OMe)-FMK, z-VAD), 1 h before DMSO or SFN (15 μM) exposure, and the whole cell lysates obtained at 24 h were immunoblotted for HDAC3, HDAC6, PARP and caspase-3. CTR, control.

PARP cleavage, which is indicative of caspase-mediated apoptosis, provided a possible mechanistic explanation for the loss of HDAC protein expression in response to SFN treatment. Specifically, HDAC3 is a reported substrate of caspase-3 [39]. However, under conditions in which both PARP and caspase-3 were cleaved, SFN-induced loss of HDAC3 was not associated with the appearance of an HDAC3 cleavage product (Figure 5A). Time-course SFN studies revealed the near simultaneous loss of full-length HDAC3 using antibodies to either the N-terminal or C-terminal portion of the protein (Figure 5B). Low molecular weight bands were detected occasionally, but these bands did not increase with the loss of full-length HDAC3, and no cytoplasmic relocalization of cleaved HDAC3 [39] was observed (data not shown). Finally, the cell-permeable pan caspase inhibitor z-VAD(OMe)-FMK blocked PARP and caspase-3 cleavage at 24 h, but did not reverse the SFN-induced loss of HDAC3 (or HDAC6) protein expression (Figure 5C). Our interpretation was that caspase-mediated HDAC cleavage did not explain the loss of HDAC protein expression in colon cancer cells treated with SFN.

### SFN-induced loss of HDAC3 is unaffected by selected proteasome and lysosome inhibitors, but is attenuated by cycloheximide and actinomycin D

**Figure 6:**
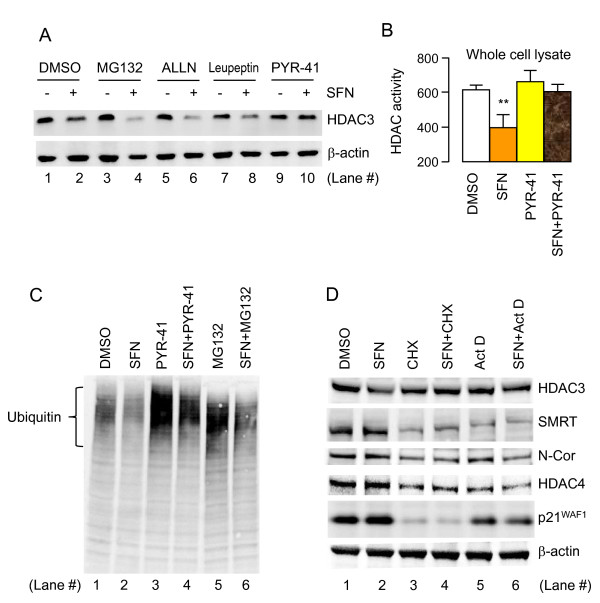
**Probing the pathways of protein turnover and stability in SFN-treated colon cancer cells**. (A) HCT116 cells were treated with MG132, *N*-acetyl-Leu-Leu-norleucinal (ALLN), leupeptin, or PYR-41 [47], in the presence and absence of SFN (15 μM). Whole cell lysates obtained at 24 h were immunoblotted for HDAC3. For the concentrations of each inhibitor, see Methods. (B) HDAC activity in the whole cell lysates obtained at 24 h from HCT116 cells treated with DMSO, SFN, PYR-41 (PYR), or SFN+PYR. Data (mean ± SE, n = 3); **P < 0.01 *versus *the DMSO control. (C) HCT116 cells were treated with inhibitors, as shown, and the whole cell lysates were immunoblotted at 24 h for total cellular ubiquitin. (D) HCT116 cells were treated with cycloheximide (CHX), actinomycin D (Act D), SFN, SFN+CHX, or SFN+Act D. Whole cell lysates were immunoblotted at 6 h for HDAC3, HDAC4, SMRT, N-Cor and p21^WAF1^. Data are representative of findings from two or more separate experiments.

Following the caspase studies, subsequent experiments assessed mRNA transcript levels *via *quantitative real-time PCR, for class I and class II *HDAC*s. No concordance was seen with respect to SFN-induced changes in HDAC protein expression (data not presented). Next, selected inhibitors were used to probe different pathways of protein turnover and stability. Proteasome inhibitor MG132, calpain inhibitor *N*-acetyl-Leu-Leu-norleucinal (ALLN), and protease inhibitor leupeptin did not block the SFN-induced loss of HDAC3 protein expression (Figure 6A). On the contrary, loss of HDAC3 was enhanced when SFN was combined with these inhibitors. Prior reports described the synergistic interactions between HDAC inhibitors and proteasome inhibitors [43-46]. PYR-41, a purported inhibitor of the E1 ubiquitin-activating enzyme [47], blocked the SFN-induced loss of HDAC3 protein expression (Figure 6A, lanes 9 and 10). HDAC activities in the corresponding PYR and PYR+SFN whole cell lysates were identical to the vehicle control (Figure 6B).

Total cell lysates next were probed with an anti-ubiquitin antibody (Figure 6C). High-molecular weight poly-ubiquitylated bands were detected in the vehicle controls (lane 1), and these bands were reduced by SFN treatment (lane 2). In contrast, PYR-41 produced a striking increase in poly-ubiquitylated bands (lane 3), over and above those that accumulated in response to MG132 treatment (lane 5). SFN co-treatment partially overcame the increased poly-ubiquitylation associated with either PYR-41 or MG132 (Figure 6 C, compare lane 4 *versus *lane 3, and lane 6 *versus *lane 5).

As noted in the introduction, regulation of p21^WAF1 ^in colon cancer cells has been associated with a corepressor complex involving HDAC3-HDAC4-SMRT/N-CoR [18]. Treatment with cycloheximide (CHX) for 6 h, in the presence or absence of SFN, depleted SMRT, N-Cor and HDAC4, as well as p21^WAF1^, but had little or no effect on HDAC3 expression (Figure 6D, lanes 3 and 4). Similar results were obtained with Actinomycin D, in the presence or absence or SFN, although the loss of p21^WAF1 ^was less marked (Figure 6D, lanes 5 and 6). These data supported the view that HDAC3 protein was relatively stable in HCT116 cells, whereas SMRT, N-Cor, and HDAC4 (as well as p21^WAF1^) had a shorter half-life. On the other hand, SFN treatment reduced HDAC3 protein expression at 6 h without attenuating SMRT, N-Cor, or HDAC4. Notably, the SFN-induced loss of HDAC3 protein (lane 2) was fully or partially blocked by CHX (lane 4) and Actinomycin D treatment (lane 6), respectively. These findings implicated one or more protein partner(s) with a relatively short half-life in the HDAC3 turnover mechanism triggered by SFN.

### Role of 14-3-3 and Pin1 in the SFN-induced loss of HDAC3

**Figure 7:**
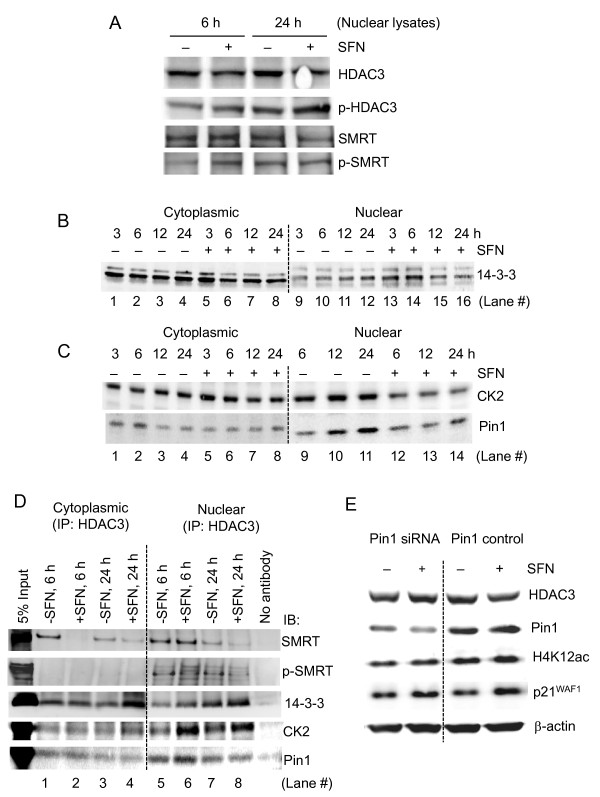
**Role of CK2, 14-3-3 and Pin1 in the mechanism of SFN-induced HDAC3 protein loss**. (A) Nuclear extracts from SFN-treated HCT116 cells were immunoblotted for phospho-HDAC3 (p-HDAC3), phospho-SMRT (p-SMRT), HDAC3, and SMRT. (B,C) Time-course of 14-3-3, CK2, and Pin1 protein expression changes in cytoplasmic and nuclear extracts of HCT116 cells, normalized to β-actin (not shown). (D) Immunoprecipitation (IP) studies, pulling-down HDAC3 from cytoplasmic and nuclear extracts of HCT116 cells followed by immunoblotting (IB) for SMRT, p-SMRT, 14-3-3, Pin1, and (not shown) HDAC3. (E) siRNA-mediated knockdown of Pin1, compared to scrambled siRNA control. Cells were transfected with siRNAs, 24 h later SFN (15 μM) was added, and whole cell lysates were immunoblotted 16 h thereafter for HDAC3, Pin1, H4K12ac, and p21^WAF1^.

Previous work established that phosphorylation of SMRT/N-Cor and HDAC4 resulted in disassembly of the corepressor complexes, followed by their nuclear export and binding to 14-3-3 [48,49]. Using phospho-specific antibodies, phospho-HDAC3 (p-HDAC3) and phospho-SMRT (p-SMRT) were increased in the nucleus at 6 h and 24 h after SFN treatment, relative to total HDAC3 and total SMRT (Figure 7A). No such changes were detected for N-Cor or HDAC4 under these conditions (data not shown).

As expected, 14-3-3 levels were higher in the cytoplasm than in the nucleus, but time-course studies indicated a partial shift of 14-3-3 to the nucleus following SFN exposure (Figure 7B). Thus, whereas cytoplasmic 14-3-3 expression remained relatively constant in the -SFN controls (lanes 1-4), SFN treatment led to reductions in cytoplasmic 14-3-3, most notably at 6 h (lane 6), and there was a corresponding increase in nuclear 14-3-3 (lane 14). Two other SMRT partners were decreased in the nucleus (Figure 7C), namely protein kinase CK2 (casein kinase II) and peptidyl-prolyl cis/trans isomerase 1 (Pin1). CK2, which phosphorylates SMRT and has a phospho-acceptor site on HDAC3 [50,51], was reduced markedly in the nucleus 6-24 h post-SFN treatment (lanes 12-14). Pin1, which negatively regulates SMRT protein stability [52], increased gradually in the nucleus in -SFN controls (lanes 9-11), but remained relatively low in SFN-treated cells (lanes 12-14). In the cytoplasm, no marked changes were detected for CK2 or Pin1 in the presence or absence of SFN (lanes 1-8).

In co-immunoprecipitation (co-IP) experiments, pulling-down HDAC3 identified SMRT as a binding partner both in the cytoplasm and nucleus (Figure 7D). SFN treatment completely blocked HDAC3/SMRT interactions in the cytoplasm at 6 h (lane 2), and attenuated these associations in the cytoplasm and nucleus at 24 h (lanes 4 and 8). Although nuclear p-SMRT was increased by SFN (Figure 7A), less nuclear p-SMRT was pulled down with HDAC3 at 6 and 24 h post-SFN exposure (lanes 6 and 8, Figure 7D). No HDAC3/p-SMRT interactions were detected in the cytoplasm. Our interpretation of these findings was that increased phosphorylation of HDAC3 and SMRT led to corepressor complex dissociation, with less SMRT and p-SMRT interacting with HDAC3 after SFN treatment. Interestingly, the increased nuclear 14-3-3 at 6 h post-SFN exposure (Figure 7B, lane 14) was paralleled by enhanced binding of 14-3-3 to HDAC3 in the nucleus (Figure 7D, lane 6), which was further augmented both in the cytoplasm and nucleus at 24 h (Figure 7D, lanes 4 and 8, respectively). In the nucleus, CK2 associations with HDAC3 increased at 6 and 24 h post-SFN treatment (lanes 6 and 8, Figure 7D), despite the lower total nuclear CK2 levels in SFN-treated cells (Figure 7C, lanes 12-14). This result suggested that SFN shifted the pool of nuclear CK2 towards HDAC3/SMRT, favoring phosphorylation and complex disassembly.

In addition to the enhanced association of 14-3-3 with HDAC3, SFN treatment also increased Pin1 interactions with HDAC3 in the nucleus at 6 h (Figure 7D, lane 6). Pin1 pull-downs confirmed SMRT and HDAC3 nuclear interactions 6 and 24 h after SFN exposure, as well as HDAC6 binding, whereas little or no HDAC1 and HDAC2 were bound to Pin1 (Additional File 1). Because Pin1 has been implicated in the degradation of several proteins, including SMRT [52], we knocked-down Pin1 in HCT116 cells (Figure 7E). Following Pin1 knockdown, the SFN-induced loss of HDAC3 was prevented, and there was reduced H4K12ac as compared with Pin1 siRNA control. Induction of p21^WAF1 ^by SFN was unaffected by Pin1 knockdown (Figure 7E).

**Figure 8:**
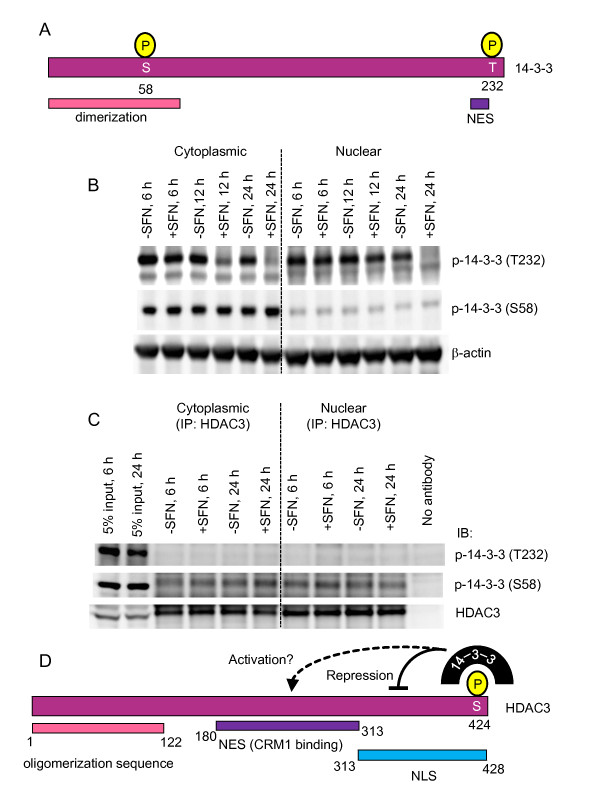
**Role of 14-3-3 phosphorylation status in HDAC3 binding**. (A) Domains in 14-3-3 showing phosphorylation sites probed by immunoblotting. (B) Nuclear and cytoplasmic extracts from HCT116 cells treated with 15 μM SFN or DMSO were immunoblotted with phosphospecific antibodies to p-14-3-3(T323) and p-14-3-3(S58). (C) HDAC3 pull-downs, performed as in Figure 7, were followed by immunoblotting for p-14-3-3(T323), p-14-3-3(S58), and HDAC3. (D) Model for 14-3-3 interacting with HDAC3: repressive actions on the nuclear localization signal (NLS) in 14-3-3, plus possible activation of the nuclear export signal (NES), are proposed based on prior studies with class IIa HDACs [56].

Finally, because the phosphorylation status of 14-3-3 can affect self-dimerization and interactions with client proteins [53,54], phosphospecific antibodies were used to probe for two such modifications (Figure 8A). Phosphorylation at T232, which negatively affects ligand binding, was lost in a time-dependent manner in cytoplasmic extracts from SFN-treated cells, and was absent in the corresponding nuclear extracts at 24 h (Figure 8B). Phosphorylation at S58 disrupts 14-3-3 dimerization and reduces the binding of some client proteins, but not all [55]. Nuclear extracts from HCT116 cells had lower basal expression of p-14-3-3(S58) than cytoplasmic extracts (Figure 8B), and these levels were unaffected by SFN treatment. Pulling-down with HDAC3 antibody and immunoblotting for p-14-3-3(T232) identified no bands, whereas p-14-3-3(S58) detected some level of interaction with HDAC3 in both the nuclear and cytoplasmic extracts (Figure 8C). In the latter case, SFN produced a slight increase in p-14-3-3(S58) at 24 h, less marked than seen with the 14-3-3 antibody used in Figure 7D (lane 4), which detects an unphosphorylated sequence conserved in the N-terminus. Based on these findings and previous studies with class IIa HDACs [56], a model is proposed for the binding of 14-3-3 to HDAC3 (Figure 8D).

## Discussion

This is the first investigation to examine the fate of individual HDACs in human colon cancer cells treated with SFN, with the dual aims of clarifying the mechanisms of the observed HDAC protein turnover and the timing of HDAC recovery following SFN removal. Pappa *et al. *[57] previously performed transient exposure experiments with SFN, observing that G_2_/M arrest and cytostatic growth inhibition were reversible in the cell line 40-16. In the present study, HCT116 cells were plated at low density so as to allow HDAC changes to be followed for at least 72 h. Under these conditions, 6-24 h of SFN exposure followed by SFN removal resulted in the complete recovery of HDAC activity and HDAC protein expression, along with the normalization of histone acetylation and p21^WAF1 ^status. Although apoptosis induction was detected, most notably at higher SFN concentrations, caspase-3-mediated cleavage of HDAC3 [39] was excluded as a contributing mechanism in the loss of HDAC3 protein. Other HDACs are known to be cleaved by caspases; for example, caspase-8-mediated cleavage of HDAC7 has been reported [40]. HDAC7 and several other class II HDACs were unaffected at the protein level by SFN treatment; however, a formal examination of each caspase and its potential HDAC target(s) may be warranted.

Changes in HDAC6 were of interest because this HDAC has been described as a master regulator of cellular responses to cytotoxic insults [42]. We performed several experiments on HDAC6 and observed the following: (i) HDAC6 protein loss was first detected at around 24 h post-SFN treatment (*versus *6 h for HDAC3); (ii) although delayed relative to other HDACs, HDAC6 was fully recovered by 72 h in the SFN reversibility studies; (iii) as with HDAC3, HDAC6 loss was not prevented by a cell-permeable pan caspase inhibitor; (iv) immunoprecipitation of HDAC3 followed by HDAC6 from whole cell lysates accounted for all of the HDAC inhibitory effects of SFN (Additional File 2); and (v) transient overexpression of HDAC6 in HCT116 cells completely blocked the increased tubulin acetylation associated with SFN treatment, as well as the induction of H4K12ac. Gibbs *et al. *[58] performed ectopic overexpression of HDAC6 in human prostate cancer cells, observing SFN-mediated inhibition of HDAC6 activity, HSP90 hyperacetylation, and destabilization of the androgen receptor. Decreased endogenous HDAC6 and HDAC3 protein expression was recently reported in SFN-treated prostate epithelial cells [59], although the precise molecular mechanisms were not pursued. We conclude that HDAC6, along with its corepressor partners, is an important target for SFN action in human prostate and colon cancer cells. However, depletion of HDAC3 followed by HDAC6 (Additional File 2), or HDAC6 followed by HDAC3 (data not shown), suggested that HDAC3 accounted for approximately two-thirds and HDAC6 one-third of the SFN actions on HDAC activity in HCT116 cells. This observation coupled with the delayed loss and slower recovery of HDAC6 compared with HDAC3 suggested that HDAC3 plays a pivotal "sentinel" role, although HDAC6 mediating HDAC3 activity probably warrants further investigation.

In the present investigation, co-IP experiments indicated that dissociation of HDAC3/SMRT corepressor complexes occurred within 6 h of SFN treatment. SMRT and N-Cor are known to be regulated by distinct kinase signaling pathways [48], resulting in corepressor complex disassembly and redistribution from the nucleus to the cytoplasmic compartment. Erk2, a mitogen-activated protein kinase, disrupts SMRT self-dimerization, releasing HDAC3 and other protein partners from the corepressor complex, thereby lowering transcriptional repression [60]. SFN is known to activate kinase signaling pathways [27,61,62], and we observed increased p-HDAC3 and p-SMRT in the nucleus within 6 h of SFN exposure, along with increased CK2 binding to HDAC3. In prior studies, phosphorylation of HDAC4 triggered its nuclear export and binding to 14-3-3 [49]. In an analogous fashion, we now report, for the first time, that there was increased binding of 14-3-3 to HDAC3 following SFN treatment. This raises the possibility that 14-3-3 sequesters HDAC3 in the cytosolic compartment, pending the subsequent release and re-entry of HDAC3 into the nucleus (e.g., upon SFN removal).

Supporting this hypothesis were the results using phosphospecific antibodies to 14-3-3. The loss of cytoplasmic and nuclear p-14-3-3(T232) upon SFN treatment is consistent with this phosphorylation impeding interactions with client proteins, such as HDAC3, and indeed no p-14-3-3(T232) was pulled down with HDAC3 in the presence or absence of SFN treatment (Figure 8C). Loss of T232 phosphorylation upon SFN treatment would provide access to the adjacent nuclear export signal in 14-3-3 [63], facilitating nuclear-cytoplasmic trafficking. On the other hand, phosphorylation of S58 in 14-3-3 shifts the pool of 14-3-3 towards more of the monomeric form, although some interaction of p-14-3-3(S58) with HDAC3 was detected. The current model (Figure 8D) proposes 14-3-3 interacting with HDAC3 phosphorylated at S424; however, other phosphorylation sites in HDAC3 may be involved, such as those associated with glycogen synthase kinase-3β [64]. Based on the findings with class IIa HDACs [56], 14-3-3 binding to HDAC3 might block the nuclear localization signal and facilitate cytoplasmic retention of HDAC3, leaving the nuclear export signal accessible to proteins such as CRM1 that further traffic HDAC3 from the nucleus to the cytoplasm. Additional work is needed to clarify this model, including the relative contributions of monomeric *versus *dimeric 14-3-3, and the role of other known phosphorylation sites in 14-3-3 [53-55].

Another interesting and novel observation was that SFN increased the binding of HDAC3 to Pin1. Pin1 knockdown completely blocked the SFN-induced loss of HDAC3, although this did not interfere with the induction of p21^WAF1^. One explanation may be that HDAC1 and HDAC2 are the primary repressor HDACs of p21^WAF1 ^[65], and neither one interacted with Pin1 before or after SFN treatment (Additional File 1). Importantly, Pin1 binding to p-SMRT has been reported to trigger SMRT degradation [52]. Proteins such as c-Myc and cyclin E use a common Pin1-interacting motif to allow turnover by the Fbw7 E3 ligase [52], but this motif does not exist in SMRT [52]. This suggests that a novel E3 ligase may be involved in the turnover of SMRT, and possibly HDAC3. There are estimated to be 500-1000 E3 ligases in human cells [47], and further work is warranted to identify the E3 ligase involved in HDAC3 turnover. Although PYR-41 has been reported as an E1 inhibitor [47], it also affects sumoylation pathways, which complicated the interpretation of PYR-41 effects on SFN-induced HDAC3 turnover in HCT116 cells. Interestingly, a selective inhibitor of CK2, 4,5,6,7-tetrabromo-2-azabenzimidazole, dose-dependently depleted Pin1 and concomitantly increased HDAC3 protein expression in HCT116 cells, further confirming the inverse association between these two proteins (P. Rajendran, data not presented).

Although the details are far from definitive and several questions remain, a model is proposed for SFN actions in human colon cancer cells (Additional File 3). Following SFN treatment, kinase signaling pathways facilitate CK2 recruitment to nuclear HDAC3/SMRT corepressor complexes resulting in the phosphorylation of HDAC3 and SMRT, complex dissociation, binding to 14-3-3 or Pin1, and trafficking from the nucleus to the cytoplasm. In the cytoplasmic compartment, sequestration of HDAC3 by 14-3-3 competes with a pathway involving Pin1-directed HDAC3 degradation. Upon SFN removal, it is postulated that HDAC3 and SMRT are released from 14-3-3 to re-enter the nucleus, reassembling the corepressor complexes to silence gene activation. Further work is needed to clarify the possible involvement of a unique E3 ligase that targets both HDAC3 and SMRT for simultaneous degradation. This model highlights the role of kinase signaling pathways triggered by SFN, but does not exclude direct actions of SFN or its metabolites on HDACs [29]. For example, entry of SFN metabolites into the HDAC3 pocket might lead to conformational changes and/or altered protein interactions that facilitate CK2 binding. These mechanisms are under further investigation in SFN-treated colon cancer cells, including time-course analyses of histone marks and the phospho-acetyl switch [66].

## Conclusions

This investigation has addressed several mechanistic questions about SFN and the HDAC changes that occur in human colon cancer cells. Despite its reported "pleiotropic" actions as a chemoprotective agent, SFN exhibited a degree of selectivity towards individual HDACs, with several class II HDACs being unaffected at the protein level. Notably, immunodepletion of HDAC3 and HDAC6, along with their corepressor partners, accounted entirely for the SFN-induced changes in HDAC activity, and cells were rescued by forced overexpression of these two HDACs. Thus, HDAC3 and HDAC6 appear to be key mediators of the transcriptional changes that occur following SFN treatment, and likely regulate the acetylation status of non-histone proteins in addition to α-tubulin, HSP90, and the androgen receptor. A novel competing pathway has been proposed involving sequestration by 14-3-3 *versus *Pin1-mediated degradation of HDAC3, but further details of the model await further study.

## Methods

### Cell culture and reagents

Human HCT116 colon cancer cells (ATCC, Manassas, VA) were cultured at 37°C, 5% CO_2 _in McCoy's 5A medium (Life Technologies, Carlsbad, CA) supplemented with 1% penicillin-streptomycin and 10% fetal bovine serum. SFN (Toronto Research Chemicals Inc. North York, ON, Canada) was prepared in DMSO and stored at a stock concentration of 10 mg/mL at -20°C. Chemical inhibitors leupeptin, ALLN, MG-132 (Sigma, St. Louis, MO) and PYR-41 (Calbiochem, San Diego, CA), were dissolved in DMSO (10 mM) and small aliquots (30 μl) were stored at -20°C. Z-VAD (OMe)-FMK was from SM Biochemicals LLC (Anaheim, CA). Cycloheximide and actinomycin D were purchased from Sigma (St. Louis, MO).

### Cell Growth

Cells in the exponential growth phase were plated at a cell density of 5,000 cells per well in 96-well tissue culture plates. After attachment overnight, cells were treated with 15 μM SFN for selected times i.e., 2, 24, 48 and 72 h. At these time points cell viability was determined using the MTT assay, as described previously [67], and cell number was counted using a Neubauer chamber.

### Flow cytometry

Cells in the exponential growth phase were plated at a cell density of 0.1 × 10^6 ^cells in 60-mm culture dishes and treated with 0 (DMSO) or 15 μM SFN. Adherent and non-adherent cells were collected at different time points i.e., 3, 6, 9, 24, 48 and 72 h in cold PBS, fixed in 70% ethanol, and stored at 4°C for at least 48 h. Fixed cells were washed with PBS once and resuspended in propidium iodide (PI)/Triton X-100 staining solution containing RNaseA. Samples were incubated in the dark for 30 min before cell cycle analysis. DNA content was detected using EPICS XL Beckman Coulter and analyses of cell distribution in the different cell cycle phases were performed using Multicycle Software (Phoenix Flow Systems, San Diego, CA).

### Cell lysates

Cells in the exponential growth phase were plated at a cell density of 0.1 × 10^6 ^cells in 60-mm culture dishes. After overnight incubation cells were treated with either 0 (DMSO) or 15 μM SFN. In some experiments a range of SFN concentrations was used (0, 10, 15, 25, 35 μM). Adherent and non-adherent cells were harvested by trypsinization at different time points, ranging from 2 to 72 h, and then washed with ice-cold PBS. Whole-cell extracts were prepared using lysis buffer containing 20 mM (pH 7.5), 150 mM NaCl, 1 mM EDTA, 1 mM EGTA, 1% Triton X-100, 2.5 mM sodium pyrophosphate, 1 mM β-glycerophosphate, 1 mM sodium orthovanadate, and 1 μg/ml leupeptin. The harvested cell pellet obtained after centrifugation was resuspended in lysis buffer and frozen at -80°C for at least 15 min, thawed on ice, vortexed for 30s and centrifuged at 13,200 × g for 5 min. To study the reversibility of SFN effects, 0.1 × 10^6 ^cells in 60-mm culture dishes were treated with DMSO or 15 μM SFN for 6 or 24 h, and the media was replaced with fresh growth medium (containing no SFN) until harvest. Whole-cell extracts were prepared at 6, 24, 48 and 72 h, and samples were frozen at -80°C until further use. Cytoplasmic and nuclear lysates were prepared using NE-PER^® ^Nuclear & cytoplasmic extraction reagent (#78833, Thermo scientific, Rockford, IL). The insoluble fraction was dissolved in SDS lysis buffer containing 65 mM Tris-HCl, pH 8.0, 2% SDS, 50 mM DTT, and 150 mM NaCl. Protease (Roche) and phosphatase (Sigma, St. Louis, MO) inhibitor cocktails were added immediately before use. Protein concentration of cell lysates was determined using the BCA assay (Pierce, Rockford, IL).

### In vitro HDAC activity

HDAC activity was measured from whole cell lysates using the Fluor-de-Lys HDAC activity assay kit (Biomol, Plymouth Meeting, PA), as reported before [68]. Incubations were performed at 37°C with 10 μg of whole-cell extracts along with the fluorescent substrate in HDAC assay buffer for 30 min. Assay developer was then added and the samples incubated at 37°C for another 30 min and read using a Spectra MaxGemini XS fluorescence plate reader (Molecular Devices), with excitation at 360 nm and emission at 460 nm. The results were expressed as AFU or AFU/μg protein.

### Immunoblotting

Equal amounts of protein (20 μg/lane) were separated by SDS-PAGE on 4-12% Bis-Tris gel or 3-8% Tris acetate gel for larger proteins (NuPAGE, Invitrogen, Carlsbad, CA) and transferred to nitrocellulose membranes (Invitrogen, Carlsbad, CA). Membranes were saturated with 2% BSA for 1 h, followed by overnight incubation at 4°C with primary antibodies against β-actin (1:50,000 Sigma, #A5441), casein kinase-IIα (1:200, Santa Cruz, #9030), cleaved caspase-3 (1:1000, Cell Signaling, #9661), acetyl histone H4K12 (1:500, Upstate, #07-595), histone H4 (1:1000, Cell Signaling, #2592), HDAC1 (1:200, Santa Cruz, #7872), HDAC2 (1:200, Santa Cruz, #7899), HDAC3 (1:200, Santa Cruz, #11417), HDAC4 (1:200, Cell Signaling, #2072), HDAC6 (1:200, Santa Cruz, #11420), HDAC8 (1:200, Santa Cruz, #11405), HDAC10 (1:200, Biovision, #3610-100), phosphoHDAC3 (1:1000, Cell Signaling, #3815), HDAC3 N-19 (1:200, Santa Cruz, #8138), N-Cor (1:1000, Abcam, #ab24552), p21^WAF1 ^(1:1000, Cell Signaling, #2947), PARP (1:1000, Cell Signaling, #9542), phosphoSMRT (pS2410, kindly provided by Dr. Marty Mayo, Univ. of Virginia, 1:1000), Pin1 (1:1000, Millipore, #07-091), SMRT (1:600, Millipore, #04-1551), acetyl α-tubulin (1:2000, Sigma, #T6793), α-tubulin (1:1000, Abcam, #ab7291), ubiquitin (1:3000, BD Pharmingen, #550944), pan14-3-3 (1:500, Santa Cruz, #629), p-14-3-3(T232) and p-14-3-3(S58), both used at 1:500 dilution (Epitomics Inc., Burlingame, CA). After washing, membranes were incubated with respective horseradish peroxidase conjugated secondary antibodies (Bio-Rad, Hercules, CA) for 1 h. Immunoreactive bands were visualized via Western Lightning Plus-ECL Enhanced Chemiluminescence Substrate (Perkin Elmer, Inc, Waltham, MA) and detected with FluorChem-8800 Chemiluminescence and Gel Imager (Alpha Innotech).

### Immunoprecipitation

HCT116 cells were treated with either DMSO or 15 μM SFN with or without pre-treatment for 1 h with PYR-41 (50 nM). Cells were harvested after 6 or 24 h and either whole cell extracts or cytoplasmic and nuclear lysates from adherent and non-adherent cells were prepared as previously described. Protein concentration was determined by BCA assay (Pierce, Rockford, IL). Protein (500 μg) was precleared with Protein A Sepharose CL-4B (Amersham Biosciences) on a rotator at 4°C for 1.5 h. Pre-cleared supernatant was collected and immunoprecipitated overnight with anti-HDAC3 (2 μg, Santa Cruz, #11417) or anti-HDAC6 (2 μg, Santa Cruz, #11420) rabbit polyclonal antibody. Protein A Sepharose beads were collected and washed before immunoblotting with anti-HDAC3 (1:200), anti-SMRT (1:500), anti-phosphoSMRT (1:700), anti-Pin1 (1 μg/ml), anti-14-3-3 (1:500), and anti-casein kinase-IIα (1:100) antibodies. The supernatant depleted of HDAC3 and/or HDAC6 was collected and kept frozen at -80°C until used for HDAC activity assays. In some experiments, HDAC3 pulls-downs were followed by immunoblotting for p-14-3-3(T232) and p-14-3-3(S58), both at 1:250 dilution.

### Overexpression and knock-down experiments

HDAC3 and HDAC6, as transfection-ready DNA in pCMV6-XL4 vector, and Pin1 siRNA (Trilencer-27) and control siRNA were from Origene (Rockville, MD). Cells were transfected using Lipofectamine 2000 (Invitrogen, Carlsbad, CA) at a ratio of 1:3-1:4 in reduced serum medium (OPTI-MEM, Invitrogen, Carlsbad, CA) according to the manufacturer's protocol. SFN treatment started after 24 h of transfection. Immunoblotting was carried out with whole cell lysates prepared using lysis buffer.

### Statistics

The results of each experiment shown are representative of at least three independent assays. Where indicated, results were expressed as mean ± standard error (mean ± SE), and differences between the groups were determined using Student's *t*-test. For multiple comparisons, ANOVA followed by the Dunnett's test was performed using GraphPad Prism. A p-value <0.05 was considered as statistically significant, and indicated as such with an asterisk (*) in the corresponding figure.

## Competing interests

The authors declare that they have no competing interests.

## Authors' contributions

PR was responsible for the reversibility and HDAC overexpression data, the immunoblotting of corepressor proteins, and Pin1 knockdown studies, plus drafting of the manuscript. BD performed time-course, reversibility, and caspase-3 cleavage experiments. WMD performed qPCR studies of class I and class II *HDAC*s, plus co-IP assays and associated immunoblotting. TWY and BW assisted with HDAC activity assays. DEW, EH, and RHD were responsible for the overall study design and interpretation of data, plus final drafting and editing of the manuscript for publication.

## Supplementary Material

Additional File 1 **Pin1 interactions with SMRT and HDACs**. Immunoprecipitation (IP) studies, pulling down Pin1 from cytoplasmic and nuclear extracts of HCT116 cells followed by immunoblotting (IB) for SMRT and HDACs 1,2,3, and 6. Click here for file

Additional File 2 **Critical roles of HDAC3 and HDAC6 in the SFN inhibitory mechanism**. HDAC activity in whole cells lysates of SFN-treated HCT116 cells, or the same whole cells lysates sequentially immunodepleted (ID) of HDAC3 followed by HDAC6. Data (mean ± SE, n = 3); **P < 0.01 *versus *the DMSO control. Similar results were obtained for HDAC6 followed by HDAC3 depletion (data not shown). Click here for file

Additional File 3 **Working model for SFN-induced HDAC3/SMRT corepressor complex disassembly, binding to 14-3-3 versus Pin1, and nuclear-cytoplasmic trafficking**. The model is discussed in the text, but several questions remain including: (i) the role of SFN *versus *its metabolites acting indirectly on kinase signaling pathways or directly on HDAC3 to facilitate CK2 binding, (ii) the nature of the 14-3-3 and Pin1 interactions with HDAC3, (iii) the effects of prolonged *versus *brief SFN exposure on HDAC3 degradation or re-import into the nucleus, and (iv) a putative novel E3 ubiquitin ligase that targets HDAC3 (and SMRT) for degradation. TF, transcription factor; HAT, histone acetyltransferase. Click here for file
