# Supplementary material for: Histone deacetylase turnover and recovery in sulforaphane-treated colon cancer cells: competing actions of 14-3-3 and Pin1 in HDAC3/SMRT corepressor complex dissociation/reassembly
Source: Mol Cancer. 2011 May 30;10:68. doi: 10.1186/1476-4598-10-68 (PMC3127849; doi:10.1186/1476-4598-10-68)
Supplement: Additional File 1 — Pin1 interactions with SMRT and HDACs. Immunoprecipitation (IP) studies, pulling down Pin1 from cytoplasmic and nuclear extracts of HCT116 cells followed by immunoblotting (IB) for SMRT and HDACs 1,2,3, and 6. [file 1476-4598-10-68-S1.PPTX]

## Slide 1
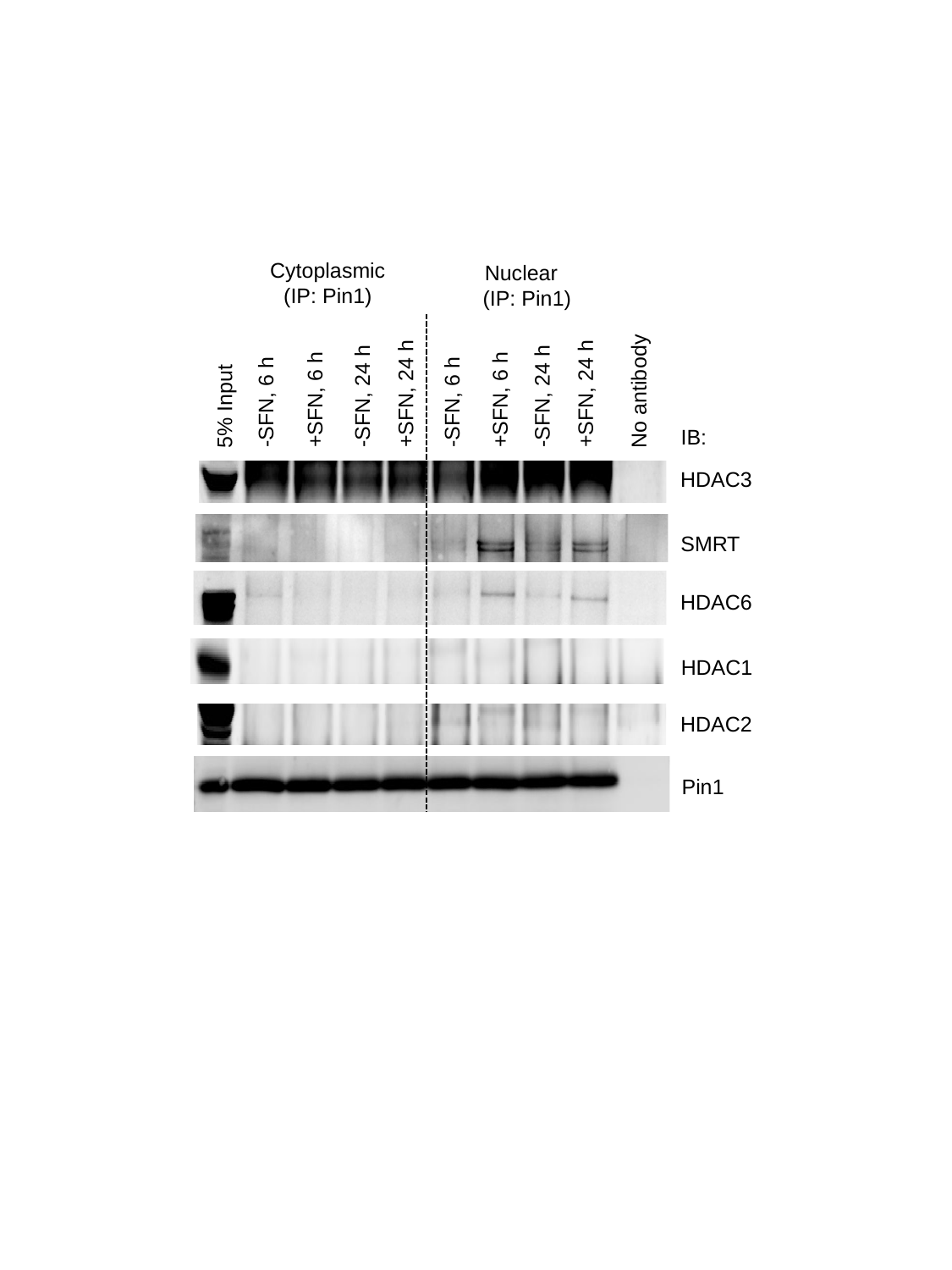

Cytoplasmic (IP: Pin1)
Nuclear (IP: Pin1)
No antibody
+SFN, 24 h
+SFN, 24 h
-SFN, 24 h
-SFN, 24 h
+SFN, 6 h
+SFN, 6 h
-SFN, 6 h
-SFN, 6 h
5% Input
IB:
HDAC3
SMRT
HDAC6
HDAC2
Pin1
HDAC1
