# Supplementary figures and images for: Histone deacetylase turnover and recovery in sulforaphane-treated colon cancer cells: competing actions of 14-3-3 and Pin1 in HDAC3/SMRT corepressor complex dissociation/reassembly
Source: Mol Cancer. 2011 May 30;10:68. doi: 10.1186/1476-4598-10-68 (PMC3127849; doi:10.1186/1476-4598-10-68)

## Slide 1
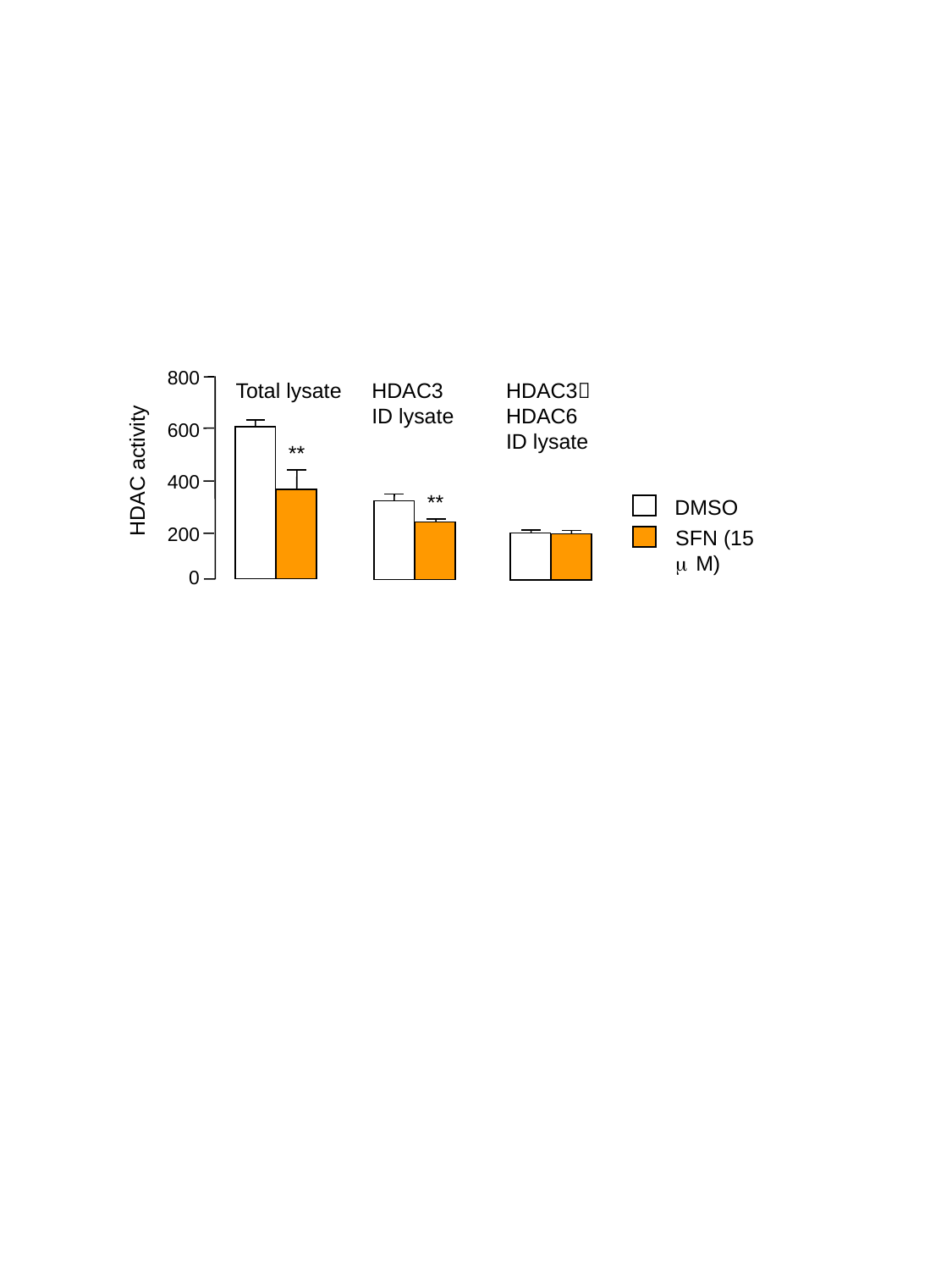

800
600
400
200
Total lysate
HDAC3 ID lysate
HDAC3HDAC6 ID lysate
**
HDAC activity
**
0
DMSO
SFN (15 mM)

Supplement: Additional File 2 — Critical roles of HDAC3 and HDAC6 in the SFN inhibitory mechanism. HDAC activity in whole cells lysates of SFN-treated HCT116 cells, or the same whole cells lysates sequentially immunodepleted (ID) of HDAC3 followed by HDAC6. Data (mean ± SE, n = 3); **P < 0.01 versus the DMSO control. Similar results were obtained for HDAC6 followed by HDAC3 depletion (data not shown). [file 1476-4598-10-68-S2.PPTX]
