# Supplementary material for: Histone deacetylase turnover and recovery in sulforaphane-treated colon cancer cells: competing actions of 14-3-3 and Pin1 in HDAC3/SMRT corepressor complex dissociation/reassembly
Source: Mol Cancer. 2011 May 30;10:68. doi: 10.1186/1476-4598-10-68 (PMC3127849; doi:10.1186/1476-4598-10-68)
Supplement: Additional File 3 — Working model for SFN-induced HDAC3/SMRT corepressor complex disassembly, binding to 14-3-3 versus Pin1, and nuclear-cytoplasmic trafficking. The model is discussed in the text, but several questions remain including: (i) the role of SFN versus its metabolites acting indirectly on kinase signaling pathways or directly on HDAC3 to facilitate CK2 binding, (ii) the nature of the 14-3-3 and Pin1 interactions with HDAC3, (iii) the effects of prolonged versus brief SFN exposure on HDAC3 degradation or re-import into the nucleus, and (iv) a putative novel E3 ubiquitin ligase that targets HDAC3 (and SMRT) for degradation. TF, transcription factor; HAT, histone acetyltransferase. [file 1476-4598-10-68-S3.PPTX]

## Slide 1
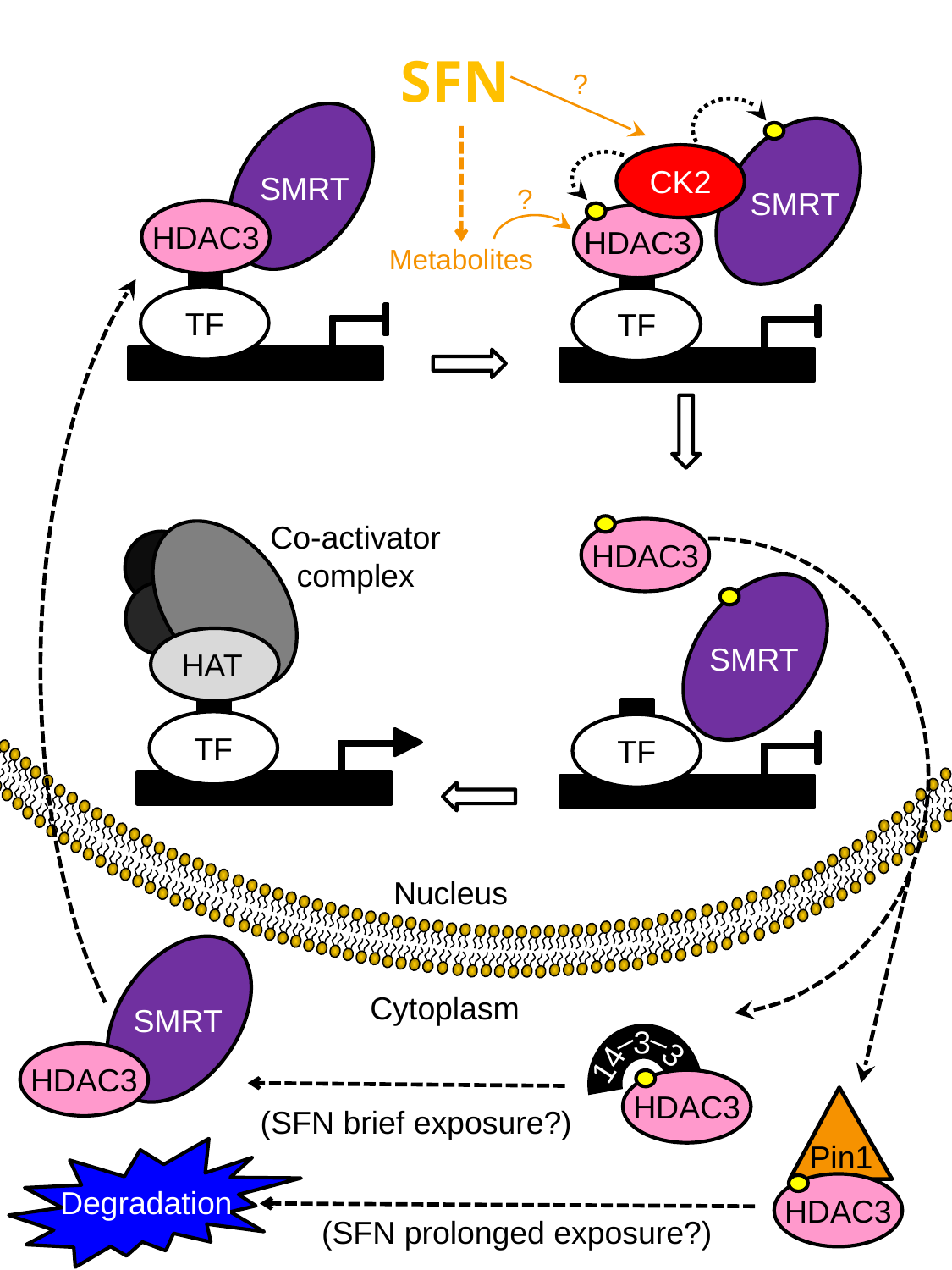

Sfn
?
SMRT
HDAC3
TF
CK2
?
SMRT
HDAC3
Metabolites
TF
HDAC3
Co-activator complex
SMRT
HAT
TF
TF
Nucleus
SMRT
 –
 –
3
3
14
Cytoplasm
HDAC3
HDAC3
Pin1
(SFN brief exposure?)
HDAC3
Degradation
(SFN prolonged exposure?)
